# Impact of Strain in Free‐Standing PtSe_2_ in Scalable 2D MEMS

**DOI:** 10.1002/adma.202412564

**Published:** 2025-08-18

**Authors:** Stefan Heiserer, Natalie Galfe, Michael Loibl, Maximilian Wagner, Oliver Hartwig, Simon Schlosser, Silke Boche, William Thornley, Nick Clark, Kangho Lee, Tanja Stimpel‐Lindner, Cormac Ó Coileáin, Josef Kiendl, Sarah J. Haigh, George J. de Coster, Georg S. Duesberg, Paul Seifert

**Affiliations:** ^1^ Institute of Physics Faculty of Electrical Power Systems and Information Technology and SENS Research Center University of the Bundeswehr Munich 85577 Neubiberg Germany; ^2^ Institute for Engineering Mechanics and Structural Analysis Faculty of Civil Engineering and Environmental Sciences University of the Bundeswehr Munich 85577 Neubiberg Germany; ^3^ Department of Materials and National Graphene Institute University of Manchester Oxford Road Manchester M13 9PL UK; ^4^ DEVCOM Army Research Laboratory 2800 Powder Mill Road Adelphi MD 20738 USA

**Keywords:** 2D material, MEMS, NEMS, piezo resistivity, PtSe_2_, strain

## Abstract

2D layered materials such as PtSe_2_ are prime candidates for next‐generation micro‐ and nano‐electro–mechanical systems (MEMS/NEMS), including piezoresistive sensors. However, due to difficulties in large‐scale synthesis and the inherent drawbacks associated with mechanical transfer of 2D material films, scalable NEMS production remains challenging. In this work, we report a fabrication route for free‐standing, as‐grown 2D material channels of PtSe_2_ with controlled dimensions, avoiding a mechanical film transfer. The free‐standing devices provide a universal platform for strain engineering of 2D materials because tension can be easily controlled by application of a back‐contact voltage. Moreover, the piezoresistivity of PtSe_2_, together with the possibility of wafer‐scale synthesis at back‐end‐of‐line compatible growth temperatures, make it ideally suited for scalable incorporation into integrated circuits. Our measurements show that the material properties can be tuned via strain, which offers pathways for classically non‐gateable materials in electronic and photonic devices. Finite element simulations of representative free‐standing films elucidate the nano–mechanical properties of large‐scale‐grown, polycrystalline 2D materials under tensile strain and demonstrate the influence of polycrystallinity on the optical and electrical behavior.

## Graphical abstract

Strain tuning of 2D materials such as PtSe_2_ opens new opportunities for micro‐ and nanoelectromechanical systems (MEMS/NEMS). This study introduces a fabrication method for free‐standing, as‐grown thin‐film channels that bypass mechanical transfer, offering a scalable, universal platform for strain engineering. Optical and electrical characterization, combined with finite‐element simulations, reveals how polycrystallinity influences the strain‐dependent properties of large‐scale‐grown 2D materials.

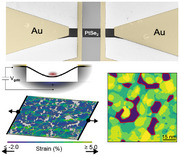

## 1 Introduction

2D van‐der‐Waals (vdW) materials are promising candidates for applications in next‐generation optical, electrical, and micromechanical devices. They have demonstrated their effectiveness in light‐emitting diodes,^[^ ^1^, ^2^ ^]^ transistors,^[^ ^3^, ^4^ ^]^ micro‐ and nano‐electro‐mechanical systems (MEMS/NEMS),^[^ ^5^ ^]^ and numerous sensors.^[^ ^6^, ^7^ ^]^ As part of their fabrication process, many devices rely on a mechanical transfer step, either from single crystals or a growth substrate onto the target substrate where the device is assembled. This dramatically impacts device yield, reproducibility, and is inherently difficult to scale given the techniques involved.^[^ ^8^, ^9^ ^]^ Even though wafer‐scale transfer of 2D materials has been reported,^[^ ^10^, ^11^ ^]^ it remains a bottleneck for real‐world devices. An alternative route is to employ direct synthesis of 2D vdW materials on target substrates, which can be achieved at wafer scale for graphene, MoS_2_, PtSe_2_, and other transition metal dichalcogenides (TMDs). These large‐scale films of TMDs are usually polycrystalline.^[^ ^12^, ^13^, ^14^, ^15^ ^]^ Therefore, they exhibit transport, optical, and mechanical properties distinct from their single‐crystal counterparts. Extensive studies have addressed the impact of polycrystallinity on 2D vdW materials’ electrical and optical properties.^[^ ^16^, ^17^, ^18^ ^]^ However, for applications in NEMS, it is necessary to have a better understanding of polycrystallinity in free‐standing devices.

In this work, we introduce a scalable fabrication technique for creating free‐standing channels from 2D materials and other thin film materials. The methodology enables us to manufacture free‐standing strain tunable channels of controlled dimensions on silicon wafers by under‐etching, without the need for mechanical transfer.

Among other materials, we apply this method to create freely suspended, devices from as‐grown PtSe_2_. PtSe_2_ is a noble metal dichalcogenide which has been demonstrated to be an excellent active material in multiple applications such as gas sensing or photonic devices.^[^ ^19^, ^20^, ^21^, ^22^, ^23^ ^]^ It exhibits strong opto–electronic interactions and undergoes a layer‐dependent transition from the semiconducting behavior in monolayers to semimetallic in the bulk.^[^ ^24^, ^25^, ^26^ ^]^ The back‐end‐of‐line (BEOL) compatible processing temperature of ≲450 °C and the wafer‐scale synthesis of homogeneous PtSe_2_ films via thermally assisted conversion (TAC) facilitate the integration of the material in conventional silicon technology, contrasting with chemical vapor deposition at higher temperatures.^[^ ^14^, ^15^ ^]^ Further, its high environmental stability^[^ ^27^ ^]^ makes this material a strong candidate for integration into real‐world devices.^[^ ^28^, ^29^, ^30^ ^]^

**Figure 1:**
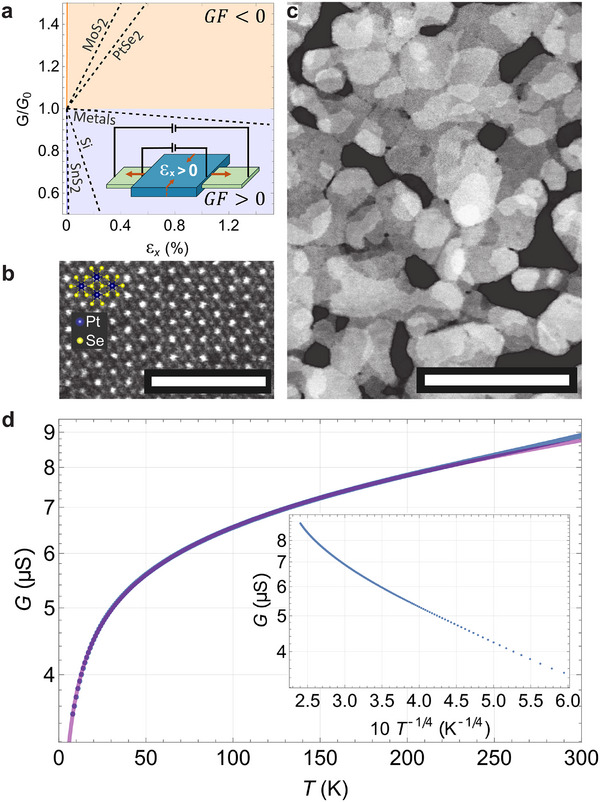
a) Conductance (*G*) versus strain of exemplary materials: Metals, Si, and SnS_2_ exhibit a positive gauge factor;^[^ ^33^, ^34^ ^]^ MoS_2_ and PtSe_2_ exhibit mostly negative gauge factors.^[^ ^19^, ^35^ ^]^ b) HAADF STEM micrograph of 4 nm thick TAC grown PtSe_2_, with a 2.5 nm scalebar and an illustrated overlay of the atomic arrangement of the 1T structure. c) A wider area HAADF STEM image with a 50 nm scalebar demonstrating the local crystallinity and global polycrystallinity of our films. d) Conductance versus temperature measurement of a 4 nm thick free‐standing PtSe_2_ that is dominated by $e^{-{\left(T_{0}/T\right)}^{1/4}}$ VRH scaling at low temperatures, as demonstrated by the inset showing the same data with adjusted axes. When fitted to Equation (1), we find *g*_0_ =  1.7 × 10^−11^ *S*, *G*_L_ =  6.3 × 10^−9^ *S* *K* ^−1^, *G_V_* =  1.1 × 10^−5^ *S*, *and* *T*_0_ =  14 K,  with *R* ^2^ =  0.999999.

PtSe_2_ exhibits a strong piezoresistivity, characterized by a significant change in its electrical resistance when subjected to tensile strain. The strain‐dependent electrical behavior opens up exciting possibilities for strain engineering in 2D FETs and its integration in NEMS devices such as pressure sensors and microphones.^[^ ^5^, ^31^, ^32^ ^]^ **Figure** **1**a shows a review of various materials used for strain sensors. A decrease in conductance with strain leads to a positive gauge factor and is observed in commercial metal strain gauges, silicon, and SnS_2_.^[^ ^33^, ^34^ ^]^ In contrast, MoS_2_ and PtSe_2_ exhibit mostly negative gauge factors as their conductance increases with strain.^[^ ^19^, ^35^ ^]^

Electrically regulated membranes can enable the voltage‐mediated application of strain to control opto–electronic properties, even in films with high carrier concentrations where field effect gating is weak, making them attractive not only for fundamental research but also for integration into strain‐sensitive transducers, pressure sensors, and next‐generation microscale actuators. Such devices are relevant across a wide range of applications, including flexible electronics, wearable systems, and MEMS/NEMS‐based signal transduction platforms.

We investigate the thermal, vibrational, and piezoresistive responses of free‐standing PtSe_2_ and its strain‐dependent structural effects. Through resistance versus temperature measurements correlated with scanning transmission electron microscopy (STEM) characterization, we conclude that the polycrystalline nature gives rise to variable‐range hopping (VRH) dominated transport behavior in thin films of PtSe_2_. Then, through finite element (FE) simulations on polycrystalline films, we provide a framework that allows us to better understand the experimentally observed piezoresistivity and strain‐dependent Raman spectroscopy in such systems. Overall, our approach to produce transfer‐free, scalable NEMS represents a universal platform to access micromechanical behavior and to modify the opto–electronic response of as‐grown 2D materials. While this approach is applicable to a broad range of 2D systems, it is particularly effective when implemented with PtSe_2_ due to its combination of strong piezoresistivity, BEOL‐compatible processing, and ambient stability.

## 2 Device Fabrication

Large‐scale PtSe_2_ films can be synthesized via TAC, wherein a pre‐deposited platinum layer is converted into PtSe_2_ in a selenium‐rich atmosphere.^[^ ^36^ ^]^ High angle annular dark field (HAADF) STEM images (Figure 1c) show the morphology of a nominally 4 nm thick film, with crystallites of different thickness overlapping to form a self‐supporting, nano‐porous network. The typical 1T stacking within the crystalline PtSe_2_ grains is experimentally confirmed at high magnification (HAADF STEM, Figure 1b).^[^ ^37^ ^]^

**Figure 2:**
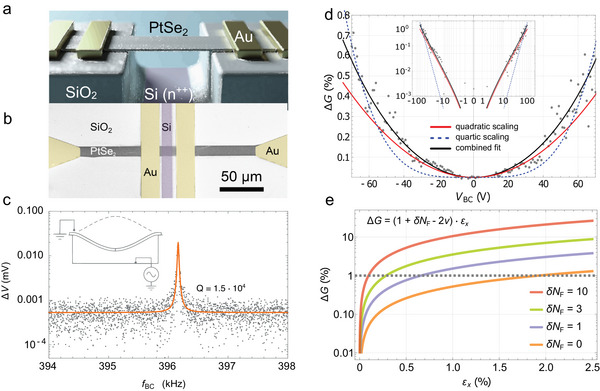
a) Schematic and b) false‐color SEM image of a free‐standing PtSe_2_ film. The SiO_2_ layer has a height of ≈ 1 µm; the bridge dimension is defined as 10 µm × 10 µm. c) An AC back‐contact voltage excites oscillations of the membrane with a resonance peak at 396.16 kHz and a quality factor *Q* of 1.5 × 10^4^. d) Change in conductance under increasing strain at 6.8 K. The conductance increases with back‐contact voltage, showing a quadratic (solid red line) and a quartic (dashed blue line) contribution. The black line illustrates the combined fit. The inset shows the data and fits on log‐log scale. e) A series of plots showing the conductance modulation predicted by the VRH scaling analysis (summarized in the piezoresistive gauge factor in Equation (4)). The graphs show how increasing density of states contributions δ*N*_F_, in turn, permit smaller tensile strains ε_*x*_ to reach Δ*G*  =  1% as indicated by the grey dashed line.

Free‐standing bridges were fabricated without mechanical transfer of the PtSe_2_ films. For this, thin Pt channels defined by lithography were sputtered onto a silicon coupon, with an ≈ 1000 nm thick SiO_2_ layer. The Pt was converted via TAC into PtSe_2_ at a BEOL‐compatible temperature of 450 °C, making large‐scale integration with CMOS devices possible.^[^ ^22^ ^]^ In‐depth material characterization of TAC‐derived PtSe_2_ was performed via time of flight secondary ion mass spectroscopy (ToF‐SIMS) and X‐ray photoelectron spectroscopy (XPS) (Figure S3a–c, Supporting Information) to demonstrate the chemical composition and successful synthesis. Further, scanning electron diffraction data (Figure S3d–f, Supporting Information) illustrate the rotational alignment of domains in the polycrystalline film. The film thickness was controlled via the thickness of the initial Pt layer and measured by atomic force microscopy (AFM) (Figures S1c and S4, Supporting Information). Four Ni/Au electrodes defined by lithography were evaporated onto the PtSe_2_ channel for electrical probing. As the final fabrication step, a photolithographically defined trench, perpendicular to the PtSe_2_ channel, was wet‐etched selectively into the SiO_2_. A buffered hydrofluoric acid (BHF) etchant intercalated between the SiO_2_–PtSe_2_ interface, under‐etching the PtSe_2_; thus, resulting in a free‐standing channel between the electrical contacts, as depicted in **Figure** **2**a.

Critical‐point‐drying in CO_2_ was employed to carefully dry the structures. A variety of free‐standing bridges with predefined widths, lengths, and thicknesses were successfully fabricated. Figure 2a shows a schematic illustration of the structure, and Figure 2b shows a top‐view scanning electron microscopy (SEM) image of the real device. A side‐view SEM image of the tilted device and an optical micrograph of an 8×8 mm^2^ chip containing 12 individual devices is shown in Figure S1. Each of the structures was screened via both optical microscopy and SEM to determine the yield of bridges that were freely suspended and not collapsed. We estimate a yield of ∼20%, which we consider as relatively high for this new fabrication route, given the nanoscale thickness of the features. The yield largely depends on the thickness of the films, as thinner films tend to become more fragile. This reflects the general trade‐off between mechanical robustness and piezoresistive sensitivity: thinner films offer enhanced electromechanical response but reduced structural stability. However, the yield could be further improved by optimizing the processing.

We were also able to demonstrate the versatility of this fabrication method and its compatibility with a broad range of material classes; we also applied our novel fabrication route to create free‐standing thin films of MoS_2_ and glassy carbon. In contrast to the semi‐metallic PtSe_2_, MoS_2_ is a heavily researched semiconductor.^[^ ^38^ ^]^ Glassy carbon, derived from pyrolysis of polymer films, is metallic and not a 2D layered material, thereby extending the applicability of our approach beyond van der Waals materials. Figure S2 illustrates optical and SEM images, as well as Raman spectroscopy of the free‐standing MoS_2_ and glassy carbon films, demonstrating the successful device fabrication. Details on the synthesis of the MoS_2_ and glassy carbon films can be found in the Methods section. In the main text, we focus on a 4 nm thick PtSe_2_ bridge since its high piezoresistivity and BEOL‐compatible growth conditions make PtSe_2_ the most promising candidate for integration in silicon technology; data on other thicknesses can be found in the Supplemental Information.

## 3 Polycrystallinity and Variable‐Range Hopping

To determine how polycrystallinity impacts the electronic transport properties of the PtSe_2_ films, we cooled the samples in an optical cryostat to ≈5 K and measured resistance versus temperature profiles. Our thickest films (≈10 nm) exhibited weak resistance increases with temperature, consistent with semimetallic transport behavior (see Figure S4, Supporting Information). In contrast, thinner films (e.g., 4 and 2.5 nm) consistently showed a resistance that decreased at high temperatures (or equivalently the conductance increased at high temperature, see Figure 1d), indicating that the semiconducting nature of the thin PtSe_2_ dominated the transport physics. Upon examination of the conductance versus temperature data, it is evident that a simple Arrhenius model cannot adequately describe the behavior. Instead, the conductance follows a VRH‐type $e^{-{\left(T_{0}/T\right)}^{1/4}}$ scaling at low temperatures, as can be seen in the inset of Figure 1d which shows a clear linear relationship between logarithmically scaled conductance *G* and *T* ^−1/4^. The 1/4 power indicates 3D transport, despite the thinness of the film, which is physically sensible as charge carriers must hop in all three directions to fully traverse the porous sample. In addition, 2D transport would necessitate a $e^{-{\left(T_{0}/T\right)}^{1/3}}$ dependence, which has been observed in other few layer van der Waals systems such as disordered nanoflakes of MoS_2_;^[^ ^39^, ^40^, ^41^, ^42^, ^43^, ^44^ ^]^ however, it fails to capture the observed behavior in thicker PtSe_2_ films. For intermediate‐to‐high temperatures, the conductance follows a simple linear dependence on temperature, and so, by treating the low and high temperature regimes as conductors in parallel, we arrive at the following formula for the entire temperature profile of the thinner films’ conductance, *G*(*T*):

(1) $$\begin{array}{c} G\;\left(T\right)=G_{V}\;e^{-{\left(T_{0}/T\right)}^{\frac{1}{4}}}+G_{L}T+g_{0}\; \end{array}$$

here *G*_V_, *G*_L_, and *g*_0_ are the VRH, thermal linear, and zero‐temperature conductance contributions, and the term *T*_0_ reflects the relevant temperature scale of VRH and includes contributions from the decay length of tunneling states, the phonon spectrum, and the density of states around the Fermi energy.^[^ ^45^, ^46^, ^47^, ^48^, ^49^ ^]^ The linear contribution physically reflects the competition between many different possible conductance terms at higher temperatures such as Arrhenius semiconducting channels and metallic channels. Over a limited temperature range, all of these appear linear in their first order Taylor expansion, and in Figure 1d, the data only begin to deviate from linearity in *T* at ≈290 K. To derive an exact physical model incorporating the effects of phonons and other resistance mechanisms^[^ ^50^, ^51^ ^]^ for the high‐temperature behavior, as was done for the ≈10 nm thick film in the Supporting Information, a higher temperature‐resistance sweep was needed. Based on the conductance analysis and the excellent fit of Equation (1) at low temperatures, we conclude that the network island growth observed in STEM has given way to polycrystalline, 3D VRH transport. The VRH temperature range of *T*_0_ =  14 K was determined for the 4 nm thick film (Figure 1d), demonstrating VRH dominance below this temperature. In contrast, applying a VRH model to resistance‐temperature data for 4.1 nm thick CVD‐grown PtSe_2_ nanosheets from Ma et al.^[^ ^52^ ^]^ resulted in *T*_0_ =  3.8 K, suggesting that VRH transport remains present in less disordered materials but is limited to cryogenic temperatures. Further comparison of our VRH model fit with other material systems is included in Table S1, Supporting Information.

## 4 Voltage‐Induced Strain and Conductance Modulation

By back‐contacting the silicon substrate, we can apply a voltage to the exposed silicon surface below the suspended PtSe_2_ films, to bend the films into the trench through the Coulomb interaction. Recall that for suspended bridges subjected to a back‐contact DC voltage, *V*_BC_, the pressure, *P*, exerted on the bridge is:

(2) $$\begin{array}{c} P=V_{\mathrm{BC}}^{2}\;\frac{\varepsilon_{0}}{2{\left(h-u\right)}^{2}}\approx \;V_{\mathrm{BC}}^{2}\frac{\varepsilon_{0}}{2\;h^{2}}\left(1+2\frac{u}{h}\right) \end{array}$$

where ε_0_ is the vacuum permittivity, *h* the initial height of the bridge, and *u* the local deformation. By Taylor expanding for *u* ≪ *h* and neglecting terms quadratic or higher order in *u*, we arrive at the standard expression for electrostatic force generated by small deformations.^[^ ^53^ ^]^ Given that for a linear‐elastic response, the strain is proportional to the applied force, Equation (2) implies that for small voltages, strain is proportional to *V* ^2^. As deformation is in turn dependent on strain, at larger voltages, corrections from higher‐order terms *V* ^2*n*^ for *n* > 1 dominate the strain.

To confirm the elastic mechanical behavior of our films, we first applied an AC voltage to drive resonant mechanical oscillations of the membranes. The first resonance frequency (fundamental harmonic) of a 10 µm long and 10 µm wide PtSe_2_ bridge was found at 396.16 kHz, where the resonance exhibited a quality factor *Q*  ≈  1.5  × 10^4^ (Figure 2c), and higher‐order modes were observed at its multiples (see Figure S6, Supporting Information). This quality factor is comparable to reported resonators from graphene and TMD materials, making our device platform suitable for highly sensitive resonant sensors.^[^ ^53^, ^54^, ^55^, ^56^, ^57^ ^]^ However, we note, that our scalable approach did not achieve high benchmark *Q* factors that were obtained from ultra‐clean single crystalline devices such as from graphene with *Q* >  10^6^.^[^ ^58^ ^]^ The fundamental harmonic could be tuned by adjusting the length and width of the bridge according to the harmonic formula for a suspended bridge $f_{1}=\sqrt{T/4\mu L^{2}}$, where *T* is the pretension and μ is the linear density which is proportional to the bridge width and height. In Figure S5d, Supporting Information, the fundamental harmonic of a bridge of dimension 80 × 40 µm^2^ and the same thickness were found at 23.9 kHz. This strongly agrees with the 1/16 geometry‐induced reduction in *f*_1_ predicted by the harmonic formula. The strength of the resonance could be tuned via the amplitude of the AC back‐contact voltage, as shown in Figure S6, Supporting Information.

By applying a DC back‐contact voltage, we can induce a static strain in the PtSe_2_ films to determine if they possess similarly negative gauge factors as transferred films on cavities^[^ ^19^ ^]^ and films grown on polyimide.^[^ ^59^ ^]^ Sweeping both positive and negative voltages, we can eliminate the influence of gate‐dependent carrier modulation by extracting the symmetric part of the conductance, Δ*G*(*V*). In Figure 2d, we present conductance data taken at 6.8 K and see that Δ*G*(*V*) can be fitted by *c* × *V* ^2*n*^, with *c* as a positive constant, *n*  =  1 at low voltages and *n*  =  2 at larger. This implies our films have a negative gauge factor, and from Equation (2) and the change in voltage power scaling, it follows that Δ*G*(*V*) depends linearly on applied in‐plane strain ε_*x*_.

It has previously been shown that bulk PtSe_2_’s negative gauge factor is a natural consequence of the enhancement of the density of states under tensile in‐plane strain.^[^ ^19^, ^37^, ^60^ ^]^ Although the *T* ^−1/4^ exponent in the VRH model indicates we have 3D transport, the semiconducting aspect of the thinner films’ transport data indicates that the 4 nm film is not thick enough to have entered the bulk PtSe_2_ regime like the ≈ 10 nm thick film in Figure S4, Supporting Information. By performing a strain‐scaling analysis of the VRH term in Equation (1), we can determine if the density of states is still the critical material property impacting the gauge factor in thin, polycrystalline films. Following refs. [48, 61], the parameters *G*_V_ and *T*_0_ in Equation (1) can be related to the hopping distance, *R*, the density of states per unit volume, *N*_F_, and the Debye frequency, ν_D_, as:

(3) $$\begin{array}{c} \;G_{V}=e^{2}\;\frac{A}{l}R^{2}\nu_{D}N_{F} \end{array}$$

with cross‐sectional area *A* and length *l* of the material. The geometric factor *A*/*l* is the traditional factor that is responsible for a positive gauge factor to most materials. In the Supporting Information, we perform a thorough analysis of how the individual components of *G*_V_ scale with deformation of the underlying material. By incorporating our scaling analysis into Equation (3), we obtain the contribution to the gauge factor, GF, from overall linear scaling of VRH conductance with in‐plane strain. Here, GF is formally determined by the change in resistance (or inverse conductance), Δ(1/*G*), as:

(4) $$\begin{array}{c} GF=\frac{G_{0}}{\varepsilon_{x}}\;\Delta \left(\frac{1}{G}\right)\approx -\left(1+\delta N_{F}-2\nu \right) \end{array}$$

As the Poisson's ratio in PtSe_2_ has been estimated as ν ≈ 0.24 (and cannot exceed 0.5), the (1 − 2ν) term alone would produce GF  =   − 0.52. For a bridge subjected to a maximum deformation of 1 µm, we expect the film to experience strains up to ε_*x*_ ≤ 0.025 as explained in the Supporting Information. In Figure 2d, we observe conductance modulations of up to ΔG ≲ 1%, which could be accounted for by GF  =   − 0.52, provided the cryogenically cooled bridges could reach maximum deformation at the larger applied voltages. However, if thermal stiffening limits the deformation, then a non‐trivial contribution from δ*N*_F_ would be necessary to obtain a larger GF to account for the cryogenic conductance modulations. In Figure 2e, we show how increasing strengths of δ*N*_F_ would permit the film to reach a conductance modulation of 1% for correspondingly decreasing strains. The positive δ*N*_F_ contribution was calculated previously in ref. [19]. Previous studies measured large negative gauge factors at higher temperatures than those at which our RT analysis indicates VRH dominates the transport.^[^ ^21^, ^59^ ^]^ These cases are still accounted for by a strong δ*N*_F_ as conductance is always proportional to the density of states of charge carriers.

## 5 STEM‐Based Finite Element Method Simulations of Strain in Polycrystalline Thin Films

**Figure 3:**
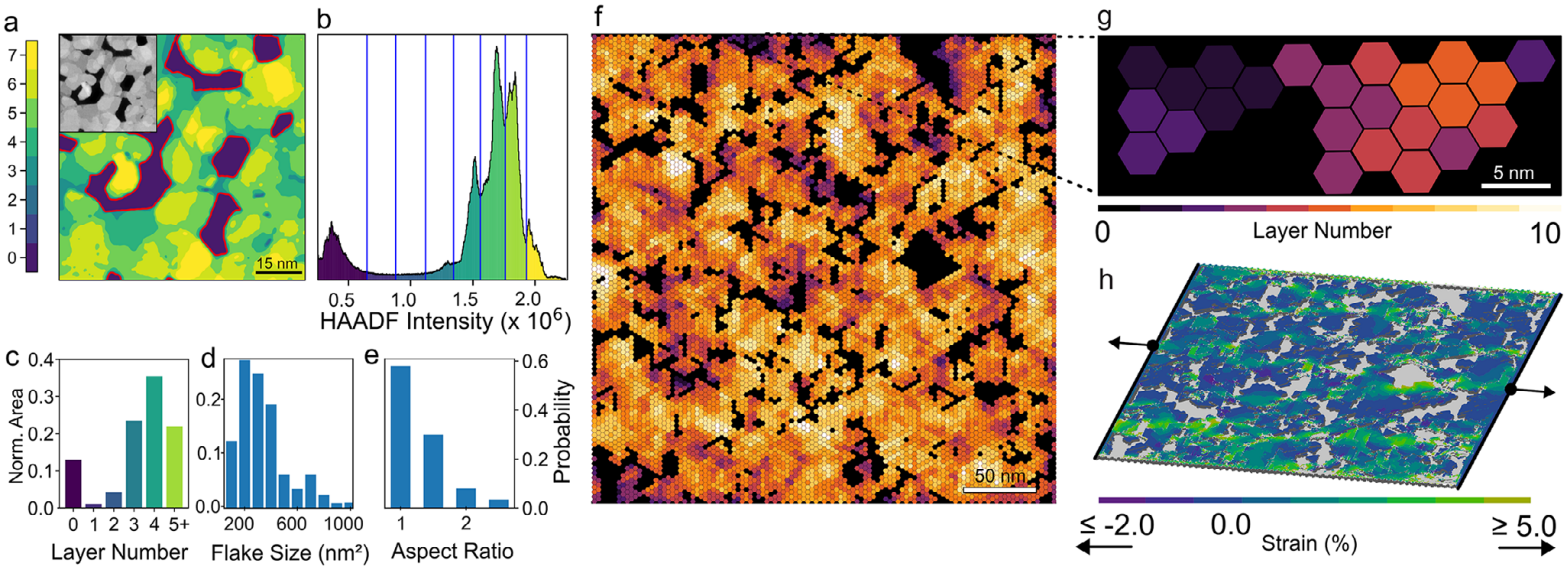
a) Segmentation of a HAADF STEM image (inset) in regions of specific layer number as assigned in the intensity histogram shown in (b). Derived statistical distribution on c) layer number, d) size, and aspect e) ratio of the crystalline grains within the PtSe_2_ film. f) FE model of a ten‐layer PtSe_2_ film of length and height 573.8 nm with porosity 0.2 and number density 0.5 (i.e., 50% of cells are filled). The film is simulated according to experimentally obtained STEM statistics. g) Close‐up showing the hexagonal unit cells within the model. Each cell has a side length of 1.8 nm and corresponds to ≈nine lattice unit cells of 1T PtSe_2_. The color indicates the number of layers for each unit cell. h) Local strain distribution of the film in (g) subjected to an overall lateral displacement of 2.5% in the *x*‐directions indicated by the black arrows. The porosity and variable thickness of the film cause the local strain to reach larger positive and weaker negative values in different patches of the film.

The electro–mechanical characteristics of the polycrystalline films fundamentally differ from the properties of their single‐crystalline counterparts. Therefore, we developed an FE model to investigate the structural behavior of polycrystalline material. The films were modeled according to the PtSe_2_ film morphology experimentally observed in STEM. We collected statistics of crystallite layer number (**Figure** **3**c), grain size (Figure 3d), and aspect ratio (Figure 3e) from experimental STEM analysis. The relative distribution of crystallite domain sizes and aspect ratios of a nominally 4 nm thick film were determined from manual measurement of two orthogonal lengths for each of 195 investigated grains. The statistics on layer number within the PtSe_2_ film were extracted from multiple HAADF STEM images, covering a total area of 30 500 nm^2^. The atomically resolved images were smoothed to average the intensity variation caused by the atomic lattice (example displayed in Figure 3a, inset). While this reduced spatial resolution, it averaged out the intensity across length scales of approximately a few unit cells, assuring the pixel intensity to be approximately linearly proportional to the corresponding film thickness. The intensity histogram shown in Figure 3b shows distinct peaks corresponding to specific number of layers. From this, the images could be divided into regions of a known thickness (Figure 3a).

We then generated randomized 3D hexagonal meshes that obeyed the experimentally measured film statistics. We used a hexagon mesh to be consistent with the hexagonal structure of the material's atomic unit cell. An example of such a mesh generation is shown in Figure 3f, where the color scale indicates the thickness of PtSe_2_, as depicted in the close‐up (Figure 3g).

An FE model was set up to perform in‐plane tensile simulations on these geometries to investigate the change of the effective thickness under strain. The FE model describes a film of ≈317 × 315 nm^2^. Its dimensions are of a similar order as the laser spot size used for the Raman spectroscopy measurements presented later, which is usually around 500 nm. Six different geometric models, which represent the statistical variety of material and void distribution retrieved from the STEM studies, were simulated. Each model consisted of ten layers where all edges were modeled without any holes to facilitate the identical application of boundary conditions across all geometries. Further details on the simulated films can be found in Table S2, Supporting Information. The boundary conditions and the imposed displacements along the *x*‐axis and *y*‐axis were adopted from the corresponding bending problem. The Supporting Information section provides a detailed discussion for treating the bending problem as a smaller‐scale tensile problem. The simulation of these smaller models, which consist of ≈310 000 elements and represent roughly one thousandth of a complete bridge, was found to be feasible within the constraints of a reasonable computational time. For simplicity, the material was assumed to have linear elasticity which seems sufficient to demonstrate qualitative behavior in this context. All strain simulations were performed in Ansys Mechanical APDL.^[^ ^62^ ^]^ A plot of the local strain distribution of a simulated structure subjected to maximum bridge deformation is shown in Figure 3h. One can observe that the porous nanocrystallinity of the film gives rise to inhomogeneity in the local strain distribution, in contrast to a single crystal film. Further details regarding the FE‐model can be found in the methods section and Supporting Information.

We investigated the impact of strain on the effective thickness, which is defined as *t_eff_* = *V*_mat_ /*A*, where *V*_mat_ is the volume sum of all material and *A* is the total area in the *xy*‐plane. Based on this definition, an effective thickness could be directly related to density, too. In the case of a homogeneous (i.e., non‐porous) material, the influence of the transverse strain could be estimated based on Hooke's law by:

(5) $$\begin{array}{c} \;\frac{t_{\mathrm{eff}}}{t_{\mathrm{eff},0}}=1+\varepsilon_{z}=1-\nu \varepsilon_{x} \end{array}$$

where *t*_eff,0_ is the initial effective thickness, for example, for a Poisson's ratio ν  =  0.24 and a strain ε_*x*_ =  0.05, the thickness change due to transverse strain would be 1.2%.

**Figure 4:**
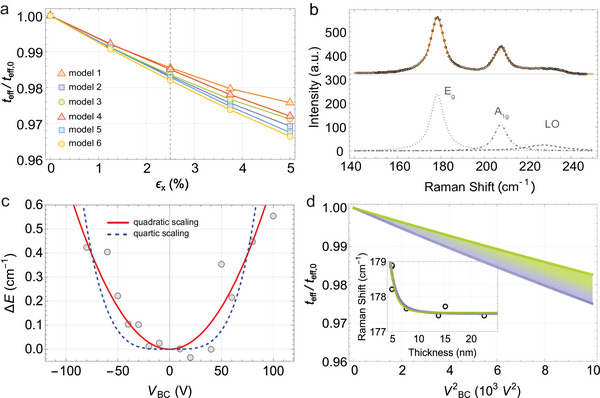
a) Simulations show a decrease of the effective film thickness under applied strain. Different films are modeled with a statistical variation of voids; details of the different models are displayed in Table S2, Supporting Information. The grey dashed line indicates a strain of 2.5%, expected under maximum bridge deformation. b) Raman spectrum of an unstrained PtSe_2_ film consisting of in‐plane *E*_g_, out‐of‐plane *A*_1g_, and longitudinal optical (LO) modes. c) The change in the *E*_g_ peak position, Δ*E*, is seen to depend quadratically on the applied back‐contact voltage, *V*_BC_. d) The inset shows the dependence of the PtSe_2_ *E*_g_ peak position on film thickness. Using the fit function from Equation (6) with 5 < *m* < 6 (respectively, blue to green shaded region), we find the relative layer thickness to decrease with back‐contact voltage (main plot). The *x*‐axis is chosen as $V_{\mathrm{BC}}^{2}$ as this is proportional to the strain in the film for small voltages. *V*_BC_ =  100 V is expected to impose near maximum deformation on the bridges, and correspondingly 2.5% strain in (a).

The results of the study are illustrated in **Figure** **4**a. The change of *t*_eff_ is plotted with respect to the strain in the *x*‐direction. The strain is not homogeneously distributed in the structure due to its porosity. Therefore, a fictitious strain value, which is computed as the imposed displacement divided by the length of the structure in *x*‐direction, is plotted to better link to the bending problem. The results show almost linear behavior due to the assumption of linear elasticity. However, the curves are not exactly linear since *t*_eff_ is a rational function of area; and therefore, of strain. The results show a maximum change of *t*_eff_ between 2.4% and 3.4% with an average of 3.0%, which is significantly higher than the expected change solely from the Poisson effect in the thickness direction. Therefore, the main contribution to the decrease of the effective layer number is directly related to the porous network and the polycrystallinity of the simulated material. The observed variation shows the statistical influence of the random hexagon mesh. In our scale analysis of the VRH conductance model, a simple homogeneous Poisson behavior is assumed for the whole material. To account for the inhomogeneous strain shown by the FE simulations, one can replace 1 − 2ν by 1 − ν_y_ − ν_z_ in Equation (4), with an out‐of‐plane effective Poisson's ratio ν_*z*_ ≈ 2.5 × 0.24. In this case 1 − ν_*y*_ − ν_*z*_ remains positive but δ*N*_F_ must have a greater contribution to produce strong gauge factors.

## 6 Strain‐Dependent Raman Spectroscopy

From the simulations presented above, we see that under applied strain, the thickness of a polycrystalline film decreases more than one would expect for a single‐crystalline film, considering Poisson's ratio of ν ≈ 0.24.^[^ ^37^, ^63^, ^64^, ^65^, ^66^ ^]^ The variation for different densities and void distributions is presented in Figure 4a. We find that the effective thickness decreases with applied strain, reaching a maximum slope of (1 − 0.955)/5%  =  0.009 per % strain.

To confirm the predicted structural changes of the PtSe_2_ film under strain, we performed room‐temperature Raman spectroscopy as this technique probes vibrational properties of the lattice. Figure 4b shows the Raman spectrum of a PtSe_2_ film without applied strain. It consists of the typical in‐plane *E*_g_ mode around 178.1 cm^−1^ (6.3 cm^−1^ FWHM), out‐of‐plane *A*_1*g*_ mode around 207.7 cm^−1^ (6.7 cm^−1^ FWHM), and a broader longitudinal optical (LO) phonon contribution as is typically observed for PtSe_2_.^[^ ^21^, ^36^ ^]^

With increasing strain, we observe a shift of the *E*_g_ peak toward higher phonon energies. Figure 4c shows the evolution of the peak center as a function of applied back‐contact voltage. For both positive and negative voltages, a blueshift of the *E*_g_ mode is observed. While the applied electric field could cause shift of the Raman mode positions due to a change in carrier concentration in the PtSe_2_, the change would not be symmetric under a positive/negative applied electric field. Therefore, the Raman shift is attributed to the physical distortion of the material due to strain. Like the strain‐dependent electrical behavior, we fit the shift of the *E*_g_ peak, Δ*E*, with both quadratic and quartic dependencies on voltage. We find quadratic dependence is sufficient to describe the behavior of Δ*E* as we would expect from Equation (2) at relatively low voltage. This, in turn, indicates that Δ*E* has a positive linear dependence on tensile in‐plane strain. This is a surprising result as *E*_g_ is an in‐plane optical phonon mode, and this parameter in single crystals conventionally decreases with tensile strain as the lattice constant is lengthened and has been well documented for other TMDs.^[^ ^67^, ^68^, ^69^, ^70^, ^71^, ^72^ ^]^ Therefore, we attribute the observed blueshift to the polycrystalline nature of our PtSe_2_ films.

We hypothesize that the observed increase in the *E*_g_ Raman mode energy under strain is due to the nano‐porous, polycrystalline nature of the films and the significant decrease of effective thickness, beyond the Poisson effect in the thickness direction. It has been shown in previous works^[^ ^21^, ^36^ ^]^ that for decreasing film thickness, the *E*_g_ mode in PtSe_2_ shifts to higher energies due to mode stiffening: The *E*_g_ mode involves in‐plane vibrations where Pt and Se atoms move parallel to the plane of the layers. In multilayers, the interlayer coupling affects how the layers slide over each other. Decreased dielectric screening and interlayer interactions in PtSe_2_ with reduced thickness increase the restoring forces for in‐plane vibrations. We can quantify the dependence of *E*_g_ on PtSe_2_ film thickness by fitting a thickness‐dependent model to the data in ref. [21], as shown in the inset of Figure 4d. We define the value of the *E*_g_ mode for infinitely thick films to be its bulk value, *E*_bulk_, and so for films of finite thickness, *t*, we fit the data with a phenomenological formula reflecting phonon mode quantum confinement:

(6) $$\begin{array}{c} E_{g}\;\left(t\right)=E_{\mathrm{bulk}}+\frac{\alpha }{t^{m}} \end{array}$$

with α a fitting parameter. When *m*, a phenomenological exponent describing how sensitively the Raman shift scales with thickness due to confinement effects, is a free parameter, the fitting procedure finds *m* ≈ 5.05, but good agreement with the data is also found for *m*  =  5 and *m*  =  6. Similarly, we can fit the *E*_g_ versus back‐contact voltage data taken in this study using the function $E_{g}\;\left(V_{\mathrm{BC}}\right)=E_{g}^{0}+\gamma \;V_{\mathrm{BC}}^{2}.$ Here, γ is a fitting parameter, and $E_{g}^{0}$ is the Raman shift at no applied voltage. This fit is shown by the red curve in Figure 4c. By combining the thickness and voltage‐dependent formulas for *E*_g_, we derive a formula relating the effective thickness, *t*_eff_, of our PtSe_2_ film of initial thickness *t*_eff,0_ =  4 nm subjected to strain in our voltage‐dependent Raman measurements:

(7) $$\begin{array}{c} \;\frac{E_{g}\left(t_{\mathrm{eff}}\right)}{E_{g}\left(\;t_{\mathrm{eff},0}=4\;\mathrm{nm}\right)}=\frac{E_{g}\left(V_{\mathrm{BC}}\right)}{E_{g}\left(\;V_{\mathrm{BC}}=0\right)} \end{array}$$

We can solve Equation (7) for *t*_eff_ and determine the relative layer thickness, *t*_eff_ /*t*_eff,0_, as a function of $V_{\mathrm{BC}}^{2}$. We plot the results of this procedure in Figure 4d for m∈[5,6] in Equation (6) and find that *t*_eff_ /*t*_eff,0_ scales with $V_{\mathrm{BC}}^{2}$, indicating linear scaling with strain following Equation (2). At a maximum applied voltage of *V*_BC_ =  100 V, we find the effective thickness of the film decreases by 1.5% to 2.5% depending on the precise value of *m* in Equation (6). As explained in the Supporting Information, at maximum deformation, the bridges should experience a strain of ≈2.5%. At this level of strain, the FE simulations indicate a 1.5–2% thickness reduction of the films, indicating that the decrease of effective thickness with strain offers a natural explanation for the blueshift of the Raman modes as seen in films of different thicknesses. Going beyond the simplest sufficient explanation, our approach can also be suitable for further studies on the impact of strain on phonon modes via, for example, electron–phonon coupling or mode stiffening.

## 7 Conclusion

We developed a new fabrication route for scalable 2D MEMS devices from as‐grown PtSe_2_ and other thin film materials without mechanical transfer. The high piezoresistivity, BEOL‐compatible growth temperature, and control over dimensions facilitated the integration of free‐standing PtSe_2_ devices into existing CMOS technologies. The conductance of the PtSe_2_ films scaled with static in‐plane tensile strain. Further, dynamic measurements showed strong geometry‐dependent mechanical resonances of the bridges with high quality factors, making the device suitable for additional sensing applications. We have demonstrated that the polycrystallinity underpins both the transport and opto–electronic properties of the material, opening opportunities to further tailor these properties. FE simulations provided a deeper understanding of polycrystalline films under strain. Their opto–electronic and micromechanical response could fundamentally differ from their single‐crystalline counterparts. The observed blueshift of the in‐plane *E*_g_ Raman mode aligned well with the simulated decrease of the effective layer number of PtSe_2_ films under strain. Depending on the application, by targeting different film morphologies of PtSe_2_, one has additional methods to control the PtSe_2_’s response to strain. The universality of the platform opens the possibility to control material properties via strain; and thus, might also be adapted to classically non‐gateable materials. We aim to explore further applications and the underlying physics using the presented device as a universal platform in future studies.

## 8 Experimental Section

### Material Synthesis

Free‐standing devices were fabricated from PtSe_2_, MoS_2_, and glassy carbon. For the TAC synthesis of PtSe_2_ and MoS_2_, the Si/SiO_2_ substrates were sputter‐coated with a thin metal layer (Pt/Mo) of defined thickness. Subsequently, the metal layers were converted into PtSe_2_ and MoS_2_ under a Se and S atmosphere, respectively. For both processes, the chalcogen carrier gas contained H_2_, facilitating the formation of H_2_Se and H_2_S, respectively. The TAC process of PtSe_2_ was performed at a BEOL compatible temperature of 450 °C for 120 min, making the material most suitable for integration in silicon technology. The MoS_2_ synthesis was performed at a temperature of 800 °C for 40 min. Glassy carbon was synthesized by spin‐coating of thin polymer films and subsequent pyrolyzation at 900 °C for 60 min.

### Raman Spectroscopy Under Strain

Raman spectra were recorded using a confocal WITec alpha 300 Raman microscope with a 1800 mm^−1^ grating and a 50× long‐working‐distance objective. The *E*_g_ and *A*_1g_ modes were fitted by a Lorentzian line shape. The gate voltage was applied using an external source measure unit to induce controllable strain within the free‐standing films during the optical measurements.

### Electron Microscopy

SEM images were acquired using a ZEISS Crossbeam 550. The acceleration voltage was set to 5.0 kV and the probe current was 100 pA. HAADF STEM images were acquired using a JEOL GrandARM 300CF microscope with an accelerating voltage of 80 kV, a convergence semiangle of 24.8 mrad, and a collection range of 68–206 (±3) mrad. The pixel dwell time was 20 µs with a probe current of 12.8 pA.

### Electrical Characterization

For electrical characterization, the samples were mounted on a cold head and wire‐bonded onto a carrier chip. Low temperature measurements were conducted in an attoDRY800 cryostat from attocube. Lock‐in detection experiments were performed using a Stanford Research SRS860 lock‐In amplifier.

### Finite Element Model

All details regarding the FE model can be found in the Supporting Information.

## Conflict of Interest

The authors declare no conflict of interest.

## Author Contributions

S.H. and N.G. contributed equally to this work. P.S., G.S.D., G.J.d.C., S.J.H., S.H., and N.G. conceived the research project and designed the experiments. S.H., N.G., S.S., O.H., S.B., and M.W. fabricated and characterized the samples. S.H. performed Raman spectroscopy experiments. S.H., G.J.d.C., and P.S. performed electrical characterization of the devices. W.T. and N.C. performed STEM measurements and the statistical analysis. M.L., J.K. and G.J.d.C. developed and performed the simulations. S.H., G.J.d.C., and P.S. analyzed the experimental data; while M.L., S.H., G.J.d.C., and P.S. interpreted the simulation results. K.L. and T.S. provided experimental and technical support. S.H., G.J.d.C., C.Ó.C., G.S.D., and P.S. wrote the manuscript with contributions from all authors.

## Supporting information



Supporting Information

## Data Availability

The data that support the findings of this study are available from the corresponding author upon reasonable request.
