# Supplementary material for: Impact of Strain in Free‐Standing PtSe2 in Scalable 2D MEMS
Source: Adv Mater. 2025 Aug 18;37(43):e12564. doi: 10.1002/adma.202412564 (PMC12574633; doi:10.1002/adma.202412564)
Supplement: Supplementary file 1 — Supporting Information [file ADMA-37-e12564-s001.pdf]

ADVANCED MATERIALS

Supporting Information

for *Adv. Mater.*, DOI 10.1002/adma.202412564

Impact of Strain in Free-Standing $PtSe_2$ in Scalable 2D MEMS

Stefan Heiserer, Natalie Galfe, Michael Loibl, Maximilian Wagner, Oliver Hartwig, Simon Schlosser, Silke Boche, William Thornley, Nick Clark, Kangho Lee, Tanja Stimpel-Lindner, Cormac Ó Coileáin, Josef Kiendl, Sarah J. Haigh, George J. de Coster, Georg S. Duesberg and Paul Seifert**

Supporting Information: Impact of Strain in Free-Standing $PtSe_2$ in Scalable 2D MEMS

Stefan Heiserer[1], Natalie Galfe[1], Michael Loibl[2], Maximilian Wagner[1], Oliver Hartwig[1], Simon Schlosser[1], Silke Boche[1], William Thornley[3], Nick Clark[3], Kangho Lee[1], Tanja Stimpel-Lindner[1], Cormac Ó Coileáin[1], Josef Kiendl[2], Sarah J. Haigh[3], George J. de Coster[1,4], Georg S. Duesberg[1], Paul Seifert[1*]*

1 Institute of Physics, Faculty of Electrical Power Systems and Information Technology and SENS Research Center, University of the Bundeswehr Munich, Neubiberg, Germany
2 Institute for Engineering Mechanics and Structural Analysis, Faculty of Civil Engineering and Environmental Sciences, University of the Bundeswehr Munich, Neubiberg, Germany
3 Department of Materials, and National Graphene Institute, University of Manchester, Manchester, UK
4 DEVCOM Army Research Laboratory, 2800 Powder Mill Road, Adelphi, MD, USA

1. Device Fabrication Scheme

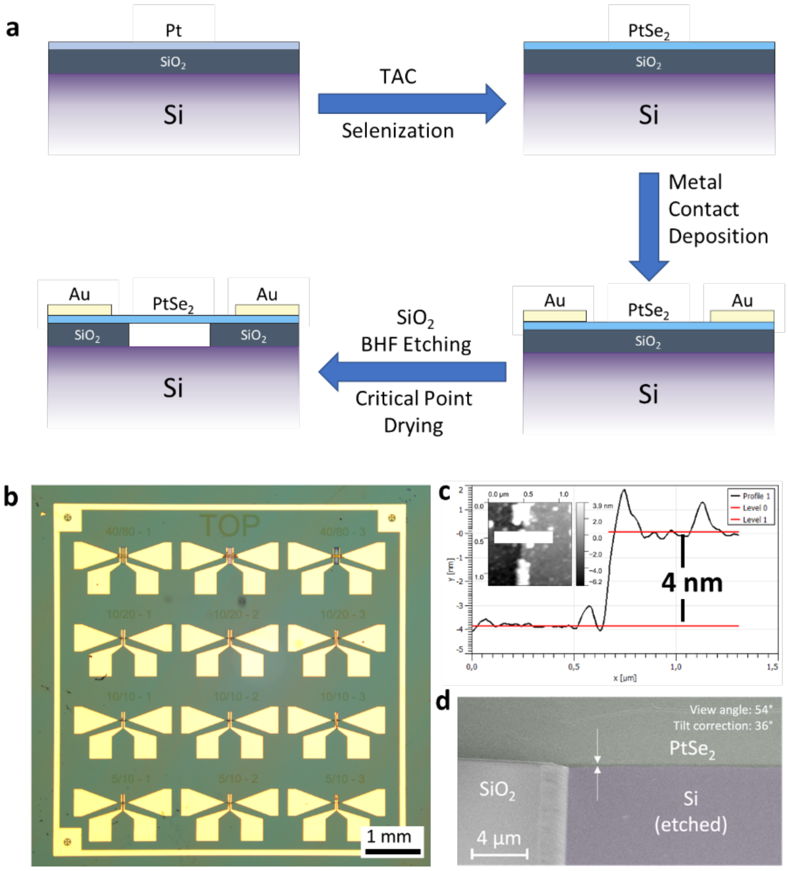

Figure S1 (a) Device Fabrication scheme for free-standing $PtSe_2$ films. Further Details can be found in the Methods section of the main paper. (b) Optical image of an 8 × 8 mm² chip containing 12 individual free-standing devices of various geometries. (c) AFM micrograph and step height profile of the 4 nm thick $PtSe_2$ film. (d) SEM image of the edge of a free-standing film under 54° viewing angle. The layers are colored for clarity.

Our device fabrication scheme enables precise dimensional control of the free-standing thin film devices, namely of the film thickness, the lateral dimensions, the height of the bridge and thereby its resonance frequency and (achievable) strain therein.
The thickness of the final $PtSe_2$ film is defined by the initial Pt layer prior to thermally assisted conversion (TAC), which is deposited via sputtering. We can fabricate films with thicknesses

ranging from ~2 nm (free-standing) to ~80 nm, enabling control over more than one order of magnitude. Thicker films offer increased mechanical stability but tend to exhibit a reduced piezoresistive gauge factor and more brittleness, whereas thinner films enhance strain sensitivity but are more fragile and prone to collapse.

The lateral dimensions of the bridge are set by pre-patterning the Pt layer using optical lithography (width) and defining the area to be under-etched in the final lithographic step (length). We have fabricated devices with lateral sizes ranging from 5 x 5 µm² to 80 x 40 µm². As shown in Fig. S5, the mechanical resonance frequencies of the bridges scale predictably with geometry, confirming the tunability of performance through dimensional design and control.

Finally, the height of the bridge is determined by the thickness of the sacrificial oxide layer (d_{ox}) prior to under-etching. This sets a physical upper limit for the strain that can be applied via electrostatic actuation: when the deformation of the bridge approaches d_{ox} the bridge risks touching the back contact. Therefore, the maximum achievable strain is limited by the geometric ratio of bridge length to d_{ox}.

2. Free-standing Devices from MoS_2 and Glassy Carbon

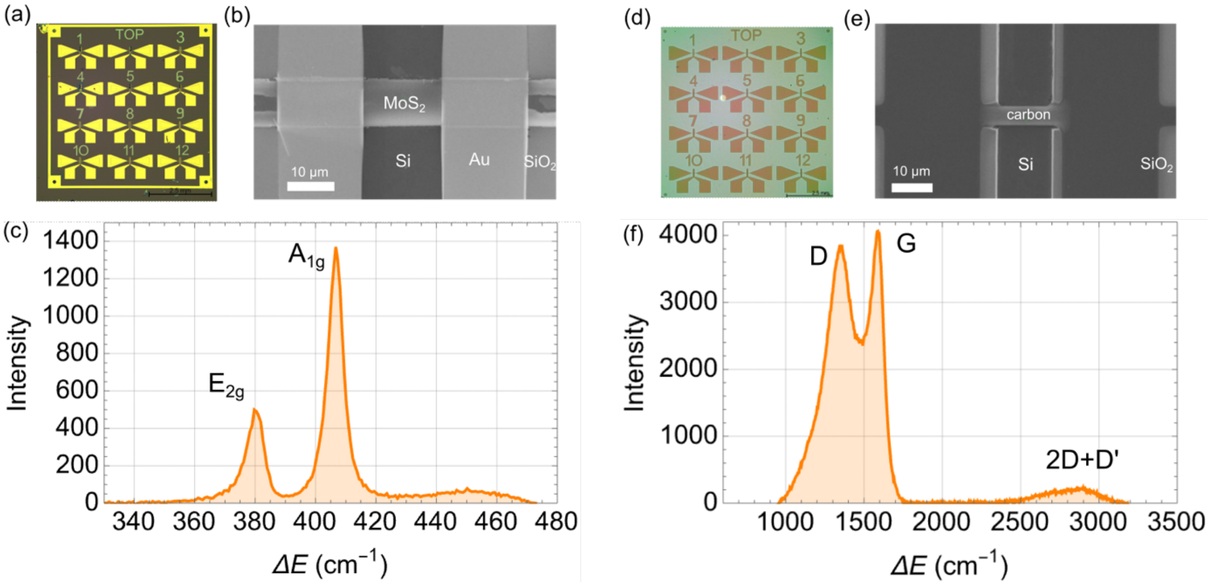

Figure S2 Free-standing devices from MoS_2 (left) and glassy carbon (right): Optical images of 8×8 mm² chips containing 12 individual free-standing devices of (a) MoS_2 and (b) glassy carbon. SEM images of free-standing (b) MoS_2 and (e) glassy carbon demonstrate the successful fabrication. Raman spectra confirm of free-standing (c) MoS_2 and (f) glassy carbon indicate the intact materials after fabrication.

3. Compositional and Structural Characterization of $PtSe_2$ Films

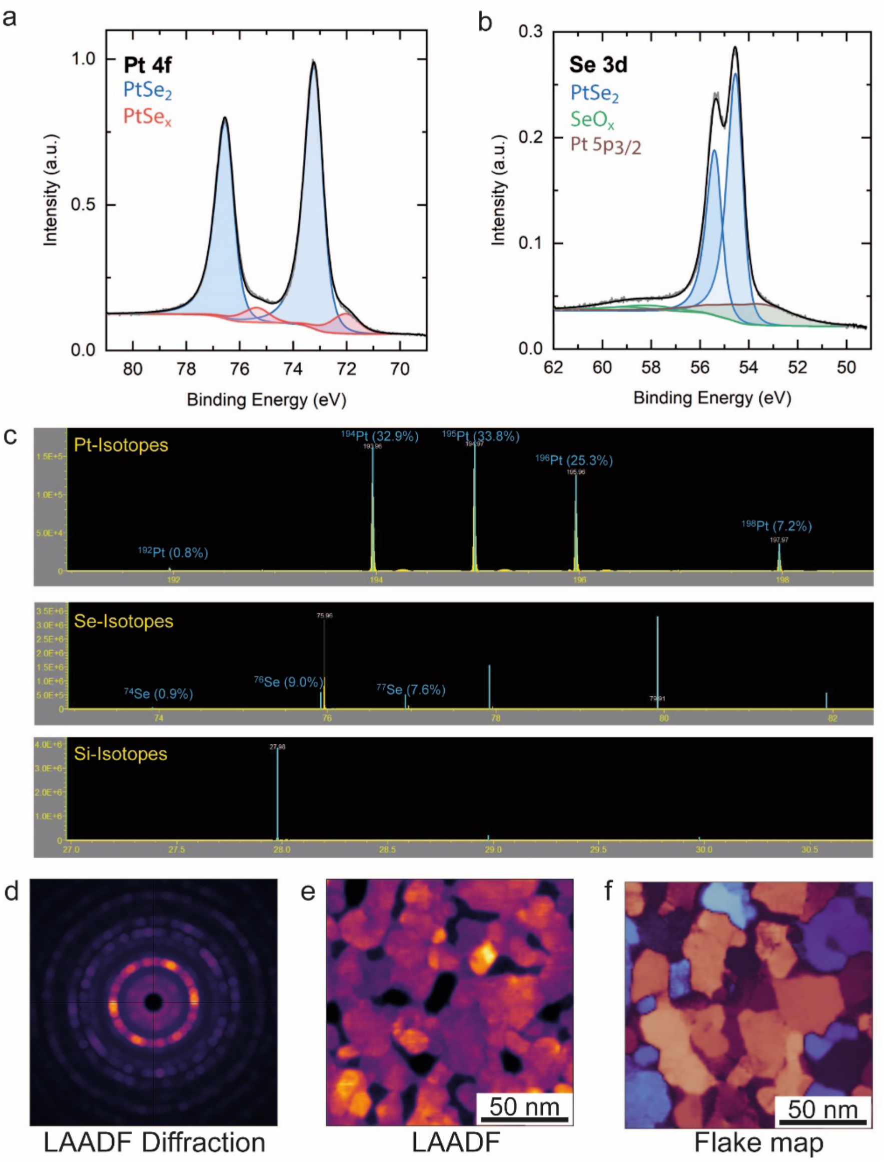

Figure S3 Chemical and structural analysis of $PtSe_2$ films: (a) and (b), XPS data of TAC-synthesized $PtSe_2$, showing the contributing Pt 4f orbital and Se 3d orbital with dominant contribution of the $PtSe_2$ bond. There is a very small contribution to the Pt 4f binding energy that comes from different stoichiometry ($PtSe_x$). For the Se 3d binding energy, there is a small contribution from selenium oxide. (c) Isotopically resolved composition of $PtSe_2$ films via ToF-SIMS. The Si contribution comes from the underlying silicon substrate. (d) 4D STEM scanning diffraction pattern showing dominant 6-fold symmetry consistent with 1T-phase $PtSe_2$. (e) LAADF STEM image of a poly-crystalline film region. (f) Angle color map of the rotational orientation of nanocrystals in the region of (e) as extracted from diffraction patterns.

4. Temperature-Dependent Conductance of $PtSe_2$ Films of Different Thickness

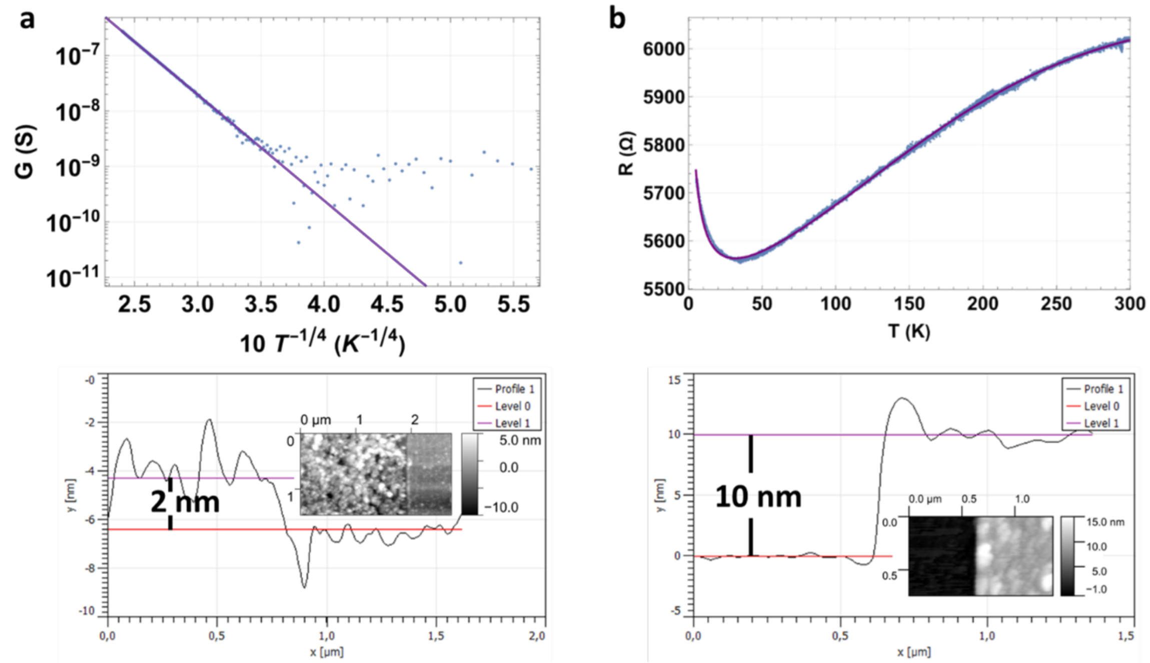

Figure S4 Temperature-dependent conductance and height profiles of a ~2 nm (a) and ~ 10 nm (b) thick $PtSe_2$ film.

For small thicknesses (e.g., 2 nm), variable-range hopping dominates up to high temperatures, which can clearly be seen from the scaled conductance versus temperature plot in Figure S4a. At low temperatures, the conductance is too small to measure in the experimental setup. In this case a pure VRH model $G(T) = G_V\, e^{-(T_0/T)^{1/4}}$ captures the data well, and we find $G_V = 1.16$ mS and a VRH temperature $T_0 = 4.01 \cdot 10^6$ K. This significantly larger VRH temperature for the 2nm film is typical of other materials where VRH persists up to room temperature.[1; 2] It accounts for the decades of conductance change in the 2 nm sample upon heating, while the 4 nm retains the same order of magnitude of conductance from cryogenic to room temperatures. This further demarks the 4 nm thickness and the transition from semiconducting to semimetallic behavior.

Conversely, films on the order of 10 nm thickness show a resistance that increases with temperature consistent with the semimetallic nature of bulk $PtSe_2$. The data for the 10 nm thick film in Figure S4b can be fitted with a resistance versus temperature model for parallel semiconducting and metallic channels that captures the crossover from metallic to semiconducting behavior as temperature increases:[3; 4]

$$R(T) = \left(\frac{1}{R_{\text{semi}}(T)} + \frac{1}{R_{\text{metal}}(T)}\right)^{-1} \qquad (S1)$$

$$R_{\text{semi}}(T) = R_\infty e^{\Delta/k_B T} \qquad (S2)$$

$$R_{\text{metal}}(T) = \left(\frac{1}{c_5 T^5} + \frac{1}{c_1 T}\right)^{-1} + \frac{c_{e-e}}{\sqrt{T}} + R_0 \qquad (S3)$$

We have defined R_{semi} and R_{metal}, the semiconducting and metallic contributions to the resistance, respectively. The parameters involved are: the infinite temperature semiconductor resistance, R_∞; the semiconducting bandgap, Δ; the zero-temperature metallic resistance, R_0; the electron-electron interaction contribution to resistance, c_{e-e} ; the low and high temperature three dimensional Bloch Gruneisen parameters, c_5 and c_1 respectively. We obtain the fit shown in purple in Figure S4 with parameter values of:

$$R_\infty = 8635\ \Omega;\ \ \Delta = 84.6\ \text{meV};\ \ c_1 = 2.74\ \Omega\ \text{K}^{-1};\ \ c_5 = 0.005\ \Omega\ \text{K}^{-5};$$
$$c_{e-e} = 975\ \Omega\ \text{K}^{1/2}; R_0 = 5304\ \Omega$$

We note that the semiconducting bandgap $\Delta = 84.6$ meV is in good agreement with theoretical calculations of 80-100 meV for the bandgap at the K-point in $PtSe_2$.[5]

5. Comparison of low-temperature variable-range hopping (VRH) transport parameters in MoS_2, graphene, and $PtSe_2$

Material	Crystal Type	Transport Direction	Transport Model	Exponent p	T_0(K)	T Range (K)	Source
MoS_2	Single (exfoliated, disordered)	In-plane	VRH (2D)	1/3	~10^5 – 10^2 (gate-tunable)	6 – 80	Xue *et al.* (2019) RSC Advances, 9, 17885-17890
MoS_2	Single (Nb-doped bulk)	In-plane	VRH (3D)	1/4	17.4	2 – 20	Park *et al.* (2015) APL, 107, 223107
Graphene (RGO)	Polycrystalline (disordered)	In-plane	ES VRH	1/2	30976 → 4225 (with less disorder)	4.2 – 100	Joung *et al.* (2012) PRB, 86, 235423
$PtSe_2$	Polycrystalline (~1.4 nm film)	In-plane	VRH → ES VRH	1/3 → 1/2	–	2 – 50	Zhang *et al.* (2020) APL, 116, 213104
$PtSe_2$/GaN	$PtSe_2$ poly film on GaN	Out-of-plane	VRH (3D)	~1/4 (assumed)	–	125 – 225	V. Janardhanam *et al.* (2023) Vacuum, 218, 112597
$PtSe_2$	4.1 nm CVD	In-plane	VRH (3D)	1/4	3.8	1 – 30	Extracted from Ma *et al.* (2022)
$PtSe_2$	4 nm TAC	In-plane	VRH (3D)	1/4	14	4 – 300	Our work
$PtSe_2$	10 nm TAC	In-plane	Parallel SC + metallic	–	982 SC energy gap	4 – 300	Our work
$PtSe_2$	2 nm TAC	In-plane	VRH (3D)	1/4	$4.01 \cdot 10^6$	65-300	Our work

Table S1: Comparison of low-temperature variable-range hopping (VRH) transport parameters in MoS_2, graphene, and $PtSe_2$. Abbreviations: VRH: Variable-Range Hopping (Mott-type); ES VRH: Efros–Shklovskii Variable-Range Hopping; T_0: Characteristic activation temperature in VRH models; p: Power-law exponent in the temperature dependence of conductivity, where conductivity $\sigma \propto \exp[-(T_0/T)^p]$; SC: semiconducting.

6. Geometry-Dependent Resonance Frequency of Free-Standing $PtSe_2$

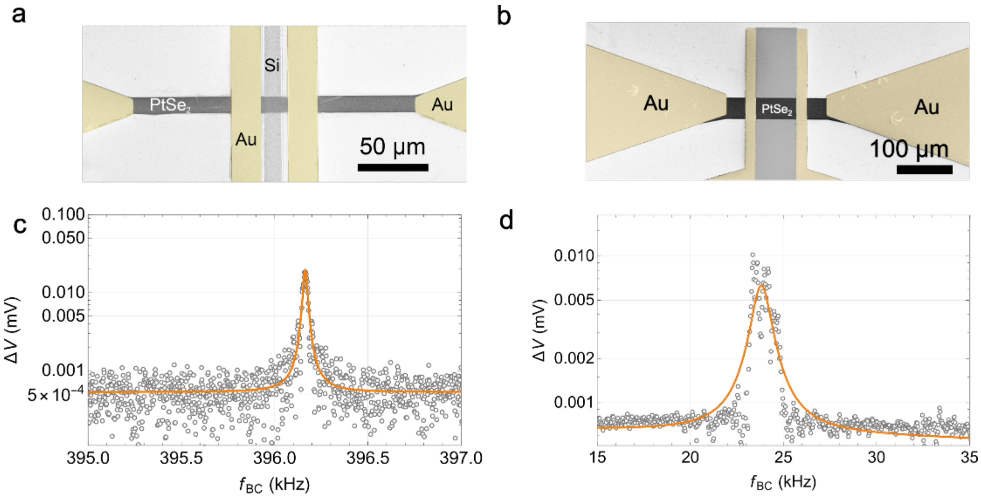

Figure S5 Comparison of the resonance frequency of devices with different geometries: SEM images of a (a) 10 × 10 µm² and (b) 40 × 80 µm² bridge. The devices exhibit a resonance at (c) 396.2 kHz and (d) 23.9 kHz, respectively. This fully agrees with the 1/16 reduction in f_1 as predicted by the harmonic formula.

7. Higher-Order and Voltage-Dependent Resonance Intensity of Free-Standing $PtSe_2$

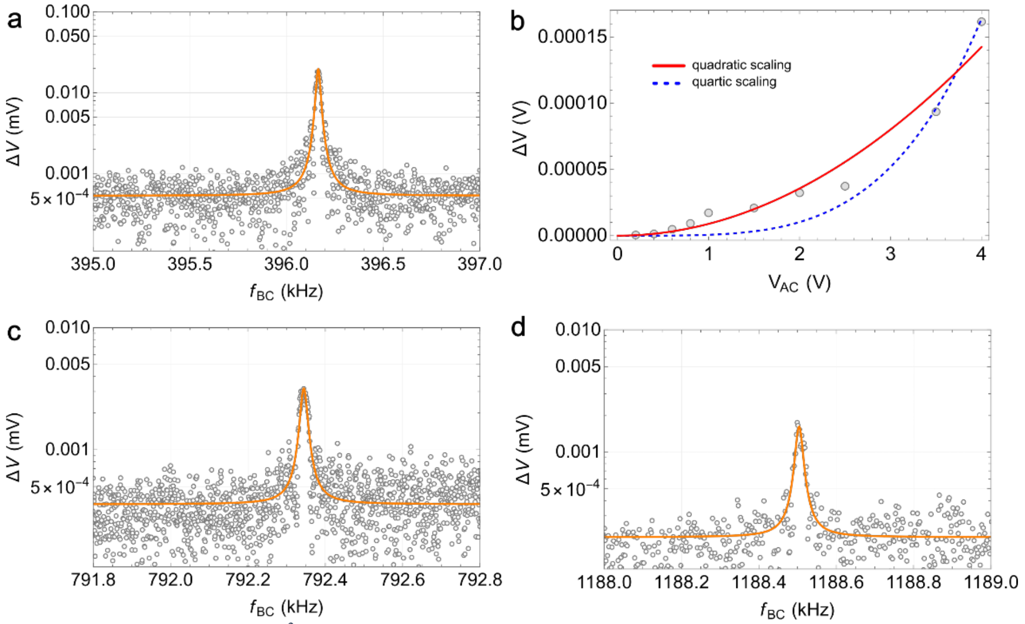

Figure S6 Resonances of a 10 × 10 µm² bridge: (a) Fundamental resonance of the free-standing device at 396.2 kHz. (b) The strength of the resonant oscillation can be tuned by the amplitude of the AC back-contact voltage. The scaling exhibits quadratic (red solid line) and quartic contributions (blue dashed line). (c) Second harmonic (792.3 kHz) and (d) third harmonic (1188.5 kHz) resonances can be found at multiples of the fundamental frequency.

8. Scaling Analysis

By performing a strain-scaling analysis of the VRH term in Eq. (1), we can determine if the density of states is still the critical material property impacting the gauge factor in thin, polycrystalline films. Following Refs. [6] and [7], the parameters G_V and T_0 in Eq. (1) can be related to the hopping distance, R, the density of states per unit volume, N_F, and the Debye frequency, ν_D, as:

$$G_V = e^2 \frac{A}{l} R^2 \nu_D N_F \,, \qquad (S4)$$

Accounting for a Poisson's ratio ν, and tensile in-plane strain ε_x the geometric factor A/l scales as $(1-\nu\varepsilon_x)^2/(1+\varepsilon_x)$, and the hopping distance in the direction of measurement R_x scales as $(1+\varepsilon_x)$. Taylor expanding and keeping only linear contributions in ε_x we obtain $(A/l)\cdot R_x^2 \sim (1+(1-2\nu)\varepsilon_x)$. The Debye frequency is insensitive to the lattice constant in the simple atomic chain model for phonons employed in the Debye model. This is because ν_D is related to material density ρ and speed of sound v_s as $\nu_D \sim \rho^{1/3}\cdot v_s$. These two terms have opposite scalings with lattice constant, and so $\nu_D \sim a^0$ [8]. Finally, let us define $\delta N_F = \frac{\partial N_f}{\partial \varepsilon_x}$, the linear variation of density of states with strain. By incorporating our scaling analysis into Eq. (S4) we obtain the overall linear scaling of VRH conductance with in-plane strain as:

$$G_V = G_0(1+\epsilon_x(1+\delta N_F - 2\nu))\,. \qquad (S5)$$

Here G_0 is the unstrained conductance. Eq. (S5) enables us to calculate the piezoresistive gauge factor, GF, when VRH is the dominant conduction mechanism, which is the case for low to intermediate temperatures as discussed following Figure 1d. In this case GF is formally determined by the change in resistance (or inverse conductance), $\Delta(1/G)$, as $GF = (G_0/\epsilon_x)\cdot \Delta(1/G) \approx -(1+\delta N_F - 2\nu)$.

9. Finite Element Model

Figure S7 depicts the 1st layer of the finite element model. The complete model consists of ten layers in total. The geometry is fully connected even though parts may not be connected within single layers but across lower or upper layers.

The model consists of multiple, equally aligned hexagons. Each hexagon unit cell of the created geometry corresponds to ~9 unit cells of 1T $PtSe_2$. Its side length is 1.8 nm and its height is 0.5nm. A layer is defined to have one hexagon in height.

The meshes – used for the results shown in Figures 3 and 4 – were created such that each hexagon was subdivided into two solid elements. The SOLID185 element of Ansys[9], which is an 8-noded linear solid element, was used. Simulations with finer meshes were performed to check that the results were sufficiently converged, but the coarser mesh was applied for the final simulations to achieve an acceptable computational time.

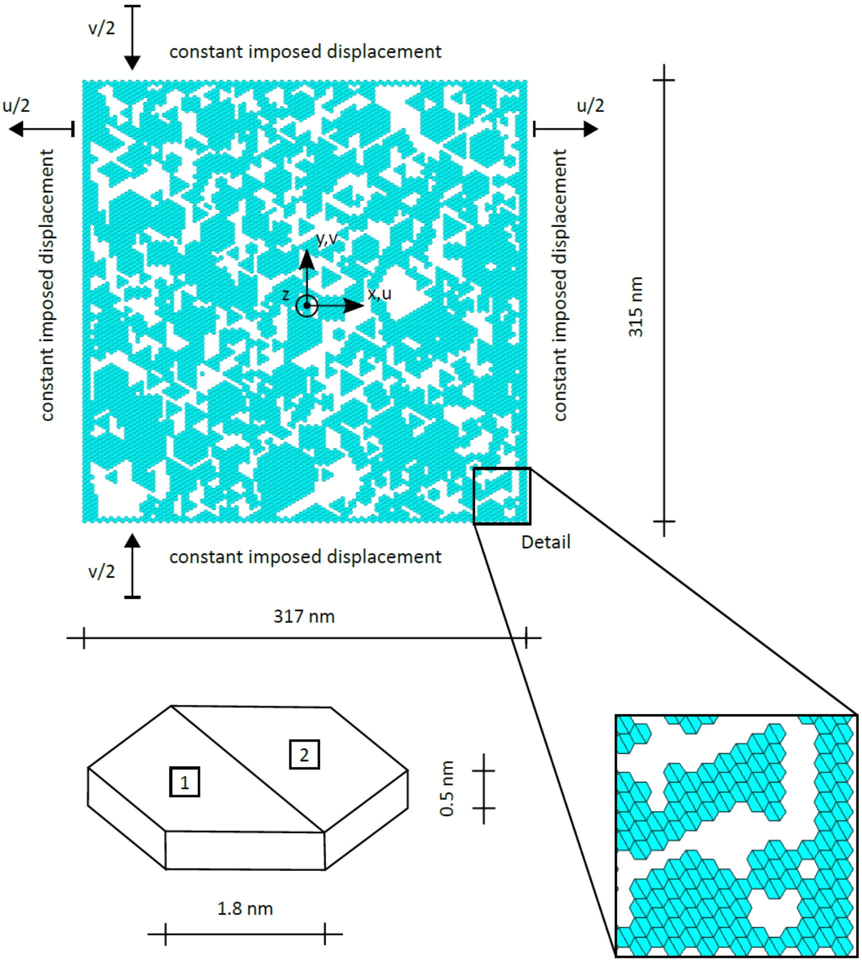

Figure S7 Finite element model setup for the 1st layer of the geometry of model 1. Each hexagon is split into two elements.

As it can be seen in the detail of Figure S7, all edges were modeled without any holes in order to facilitate an identical application of boundary conditions for all geometries. The boundary conditions are defined as: constant imposed displacements in x-direction at the left and right edge, constant imposed displacements in y-direction at the upper and lower edge, and fixed in z-direction at the 1st layer at all outer edges. The notion of edges refers to the full band of hexagons surrounding the structure. The actual values of the imposed displacements are determined in the next subsection. The boundary conditions were chosen such that they are symmetric with respect to the x-axis and y-axis and that they constrain the model as few as possible, allowing Poisson effects without being kinematic or introducing singularities due to point supports.

Table S2 presents details regarding the random geometries that are referenced by Figure 4a. The term completely-through-holes refers to holes that go through all layers, whereas the density takes all kinds of voids into account. Two different orders of densities and three different orders of completely-through-holes values were considered.

Model number	Density [%]	Completely-through-holes [%]
1	49	21
2	49	26
3	49	31
4	65	20
5	64	27
6	63	32

Table S2: Details regarding the investigated random geometries

10. Comparability of Bending and Tensile Model

The FE simulations performed in the main paper are based on a tensile model, whereas the optical measurements in the experiments are based on a bending-like deformation, as can be seen in Figure 2a. However, a tensile model was chosen for the FE simulations because modeling the complete bridge would have been too computationally expensive with the desired scale of modeled holes. Furthermore, the tensile model enabled a simpler analysis of the investigated change of thickness. In this subsection, we want to argue why the strain states for the bending and the tensile model are comparable under the assumption of elasticity. For this purpose, a simpler homogenized FE-model of the bridge was set up. The system description is presented in Figure S8a. An isogeometric geometrically non-linear Kirchhoff-Love shell was applied[10]. The maximum displacement in the middle of the bridge in bending was determined from the experiments to be approximately $1\mu\text{m}$ for a bridge of $10\mu\text{m}$ length. An elastic response in combination with large deformations was observed. Therefore, a geometrically non-linear, linear-elastic model was used. In consequence, the load-displacement relation becomes non-linear (see Figure S8b). The cross-section is completely tensioned under large deformations which is in contrast to the pure bending state expected for a linear analysis (see Figure S8c). The ratio between the mid-stress σ_{mid} and the bending part $\Delta\sigma$ is depicted in Figure S8d and shows that normal stresses dominate over bending stresses. This membrane-like behavior highly supports the tensile model used in the main part of this work. For the respective tensile model marked in Figure S8a, a reasonable choice of boundary conditions was derived from the bending model by considering the strain state under maximum deformation. The strain distributions ε_x and ε_y are depicted in Figure S8e and Figure S8f, respectively. The strain ε_x is almost constant, whereas ε_y shows the influence of the clamped edges in combination with the Poisson effect in y-direction. A detailed analysis revealed that both strains can be estimated to be constant along the small dimensions of the tensile model with a relative difference of maximum to minimum value of 0.003% and 0.012%, respectively. The extracted values for the strains under maximum displacement are $\varepsilon_x = 0.02580$ and $\varepsilon_y = -0.006059$ with a ratio between them of -4.26. These values were transferred back to displacements, which enforce the same strain state, and which were applied as boundary conditions to the tensile model. The ratio between the two normal strains was kept constant in the tensile model for simplicity even though the actual one slightly changes for the different simulated strain magnitudes (Figure 4a). The shear strain ε_{xy} is zero. In comparison, the strain ε_y for a structure which is completely free in y- and z-direction can be computed as $\varepsilon_y = -\nu\varepsilon_x = -0.24 \cdot 0.02580 = -0.006192$. This indicates that the results at midspan are almost unaffected by the clamped edges anymore. The values of the Young's modulus and the constant surface load p_z are not provided because they do not influence the presented results since only the response with respect to a given maximum displacement is of interest.

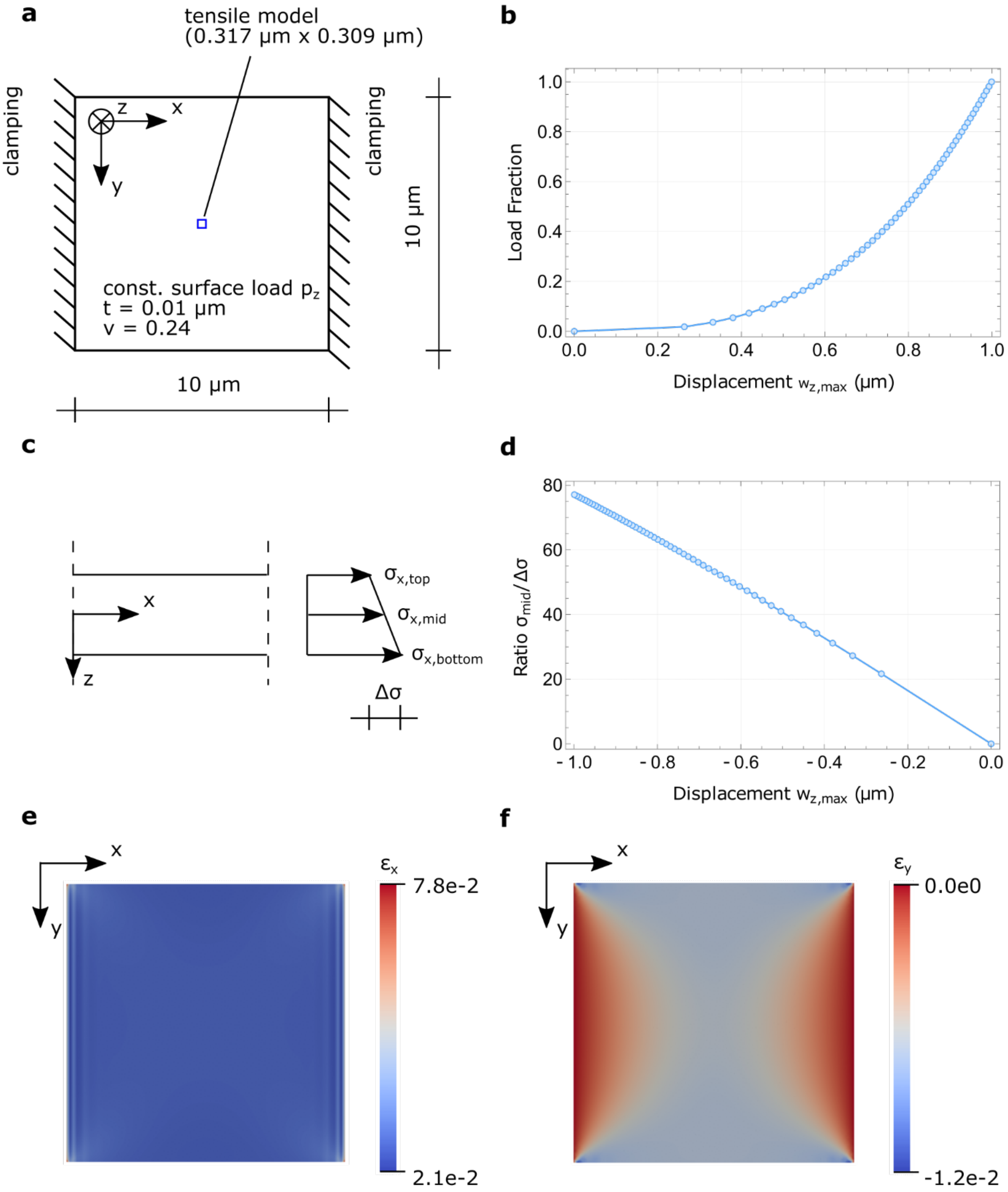

Figure S8 (a) Setup of bending problem. (b) Load-displacement curve with displacement w_{max} and load fraction. (c) Cross-sectional stress distribution in case of geometrical non-linearities. (d) Ratio $\Delta\sigma/\sigma_{mid}$ with respect to displacement w_{max}. (e) Strain distribution ε_x under maximum deformation. (f) Strain distribution ε_y under maximum deformation.

References

[1] Halim, J., Moon, E.J., Eklund, P., Rosen, J., Barsoum, M.W., and Ouisse, T., *Phys. Rev. B*, Vol. 98, 2018.

[2] Hein, P., Romstadt, T., Draber, F., Ryu, J., Böger, T., Falkenstein, A., Kim, M., and Martin, M., *Adv Elect Materials*, Vol. 11, 2025.

[3] Vail, O., Taylor, P., Folkes, P., Nichols, B., Haidet, B., Mukherjee, K., and Coster, G. de, *Physica Status Solidi (b)*, Vol. 257, 2020.

[4] Ziman, J.M., *Electrons and phonons,* OUP, Oxford, 554 p., 2001.

[5] Zhao, Y., Qiao, J., Yu, Z., Yu, P., Xu, K., Lau, S.P., Zhou, W., Liu, Z., Wang, X., Ji, W., and Chai, Y., *Advanced materials (Deerfield Beach, Fla.)*, Vol. 29, 2017.

[6] Mott, N.F., *The Philosophical Magazine: A Journal of Theoretical Experimental and Applied Physics*, Vol. 19, 835–852, 1969.

[7] Paul, D.K., and Mitra, S.S., *Phys. Rev. Lett.*, Vol. 31, 1000–1003, 1973.

[8] Ashcroft, N.W., and Mermin, N.D., *Solid state physics,* Brooks/Cole Thomson Learning, South Melbourne, 826 p., 2012.

[9] ANSYS Inc., *ANSYS Mechanical APDL 2022 R2*, https://www.ansys.com/products/structures/ansys-mechanical.

[10] Kiendl, J., Bletzinger, K.-U., Linhard, J., and Wüchner, R., *Computer Methods in Applied Mechanics and Engineering*, Vol. 198, 3902–3914, 2009.
